# Noise level in a 222 bed hospital in the 18th health region - PR.

**DOI:** 10.1016/S1808-8694(15)31073-9

**Published:** 2015-10-22

**Authors:** Marcelo Henrique Otenio, Edivaldo Cremer, Elis Marina Turini Claro

**Affiliations:** 1 Luiz Meneghel - FFALM School. Department of Health and Education - DSE.; 2 Nurse. Professor of semio-techniques.; 3 Biology Student, Environmental Health Intern.; 4 Luiz Meneghel - FFALM School. Department of Health and Education - DSE. Mailing Address: Marcelo Henrique Otenio - Embrapa Gado de Leite Juiz de Fora MG - Rua Eugênio do Nascimento 610 Bairro Dom Bosco 36038-330 Tel. (0xx32) 3249-4700 - E-mail: otenio@cnpgl.embrapa.br

**Keywords:** hospital, noise intensity, noise

## Summary

Environment noise pollution is common place today, at intolerable levels. In hospitals, technological developments have, as a consequence, potentially harmful noise levels. Much of the hospital noise comes from inside, rather than outside, and the major source of such noise is the Intensive Care Unit, for example equipment and hospital staff talk. Our goal with the present study was to investigate the noise level present in the different hospital environments, within a 222 bed hospital located at the 18th health zone, PR. Materials and Methods: The study was carried out in March, 2005, during a period of 24 hours, in tem different sectors. **Case study:** We checked environmental sound level by means of a model 1350 decibel meter. **Results:** The sound level found in our study was of 63.7 dB(A) in average, which exceeds the 45 dB recommended by the Brazilian Association of Technical Standards (1987). **Conclusion:** In the analyzed sectors, the sound level was considerably above the recommended maximum. The hospital staff should be aware of this noise level and its effects, so that they may act in a more efficient way in order to reduce this noise pollution; thus benefiting the professionals and patient recovery.

## INTRODUCTION

Environmental sound pollution, a problem that started with the industrial revolution, is today omnipresent, and intolerable. Almost nowhere is free from noise, at home, on the streets, or at work. Also in hospitals, technological progress brings, as a consequence, potentially harmful noise levels.^1^

Noise Induced Hearing Loss (NIHL) means sensorineural hearing loss, caused by the systematic occupational exposure to high sound pressure levels. A major characteristic of this loss is that it is irreversible and gradually progresses with time. Among all hearing impairments, NIHL is the most common pathology.^2^

One place that seemed free from this pollution were hospitals; nonetheless, many of them are located in areas exposed to external noise sources, such as traffic in major thoroughfares, airports, etc. However, it seems that much of the hospital noise comes from inside, rather than outside; and the major sources of noise are the Intensive Care Units (ICU - e.g. the equipment and the staff talk in there). In the ICU, there are many equipment with sonorous alarms, essential in order to alert physicians and nurses about changes in patient's conditions, or even malfunction of the devices themselves. Thus, this environment that should be quiet and calm, becomes noisy and stressful, increasing anxiety and the pain sensation, reducing sleep and prolonging hospital stay.^1^

This occupational exposure to intense noise levels is associated with many systemic manifestations, such as an increase in awareness level, increase in heart rate, changes in blood pressure and bowel movement, pupil dilation, increase in thyroid hormone production and stress.^3^

Patients in the ICU may present behavior disorders, such as the so called “ICU psychosis”, which become worse with sleep deprivation and caused by environmental conditions, continuous noise exposure among them.^4^

A calm and pleasant environment may benefit both patients and health care team alike. The health care professionals will experience less fatigue and less psychological and physiological stress; therefore, yielding patients' faster recovery.^1^

Our goal with the present investigation was to assess the noise level in the different hospital environments, in a 222 bed hospital, in the 18th Health Zone in the state of Paraná.

## MATERIALS AND METHODS

We used the internal environment of a 222 bed hospital located in the 18th Health Zone in Paraná, to carry out a study aimed at checking the environmental noise level by means of using a decibel meter manufactured by MINIPA® model - 1350 (Sound Level Meter) at the weighted measure range A = low level: 35~100 dB and set up in the following fashion: quick response at a range that covers noise levels of LO = 35~100dB. The hospital environmental noise level measures were carried out during a 24 hour period in March of 2005, during the week days, and in ten sectors; in each sector we carried out twelve measurements every hour, during 1 minute of average time, totaling 2,400 minutes of measurement.

The equipment was positioned at 1.25 meter high, set up on a tripod, at a point marked by adhesive tape on the floor, getting close to the head height of a patient in bed.

Measures were carried out according to sector sequence and respective equipment:

1. Emergency room reception: a bell sounds to open the door, telephone, television set, fan, waxing machine and staff.
2. Cast room: motor to saw casts and staff. There was no service in the night shift.
3. Neonatal ICU: Six incubators monitored by sound alarm devices, pulse oximeters, oxygen catheters, one phototherapy incubator; one microwave oven; one scale for daily weighing; six flowmeters for inhaling, aspirator and staff.
4. ICU: ten beds, ten vital sign monitors with sound warnings, ten pulse oximeters, flowmeters, secretion aspirator, door bell, telephone and computer; and staff.
5. Surgical center rooms 01, 02, 03, 04 and 05: surgical table, instruments' table, pulse oximeter, respirator, aspirator, electric drill, air conditioned, electrical scalpel, videoscope, surgical instruments box; and staff. OBS: There was no surgery in the night shift.
6. Sterilization central: one autoclave, one air compressor, one fan, one radio and a bell, and staff. In this sector there is only the day shift.
7. Pharmacy: one radio, one fan. Staff.
8. Pediatrics (Nurse Station): Pediatrics is divided in 13 rooms: cafeteria, outpatient ward, nurse station, waste, laundry, rooms 01 to 07 and recreation room. Flowmeters, radio, television set, fan, waxing machine. Staff, children and parents/tutors.
9. Kitchen: two stove exhausts, two wall exhausts, metal materials, silverware and china. Staff.
10. Laundry. Clean area: two washing machines, two centrifuges and ironing machines. Staff. Contaminated area: three washing machines and one fan. Staff.

Before starting each measuring task, the device was calibrated according to the manufacturer's instructions for standardization purposes. The measuring tasks were carried out by the investigator, with prior authorization from the hospital management; however, without the knowledge of the employees where the measuring was taking place, in order to avoid change of working habits.

The results are the arithmetical averages of the values found along the measurements and are presented by means of charts created with the Excel software for tabulation.

## DISCUSSION

The Brazilian Association of Technical Standards - Associação Brasileira de Normas Técnicas (ABNT) NBR 10152/1987 recommends 35 to 45 dB(A) as acceptable noise levels for different hospital environments.^1^

The noise level found in our study had a mean value of 63.7 dB (A), which exceeds the maximum allowed values of 45 dB recommended by the ABNT (1987)5, in agreement with the World Health Organization (1993)6 that recommends a noise level up to 40 dB (A) for the day shift and 35 dB (A) for the night shift in hospitals.

The International Labor Organization calculates that 140 million people in the world are exposed to harmful occupational noise levels. This is regrettable, since this is an avoidable cause of hearing loss.^7^

Usually, noise levels in a calm hospital must be in the range of 40 and 50 dB (A); in a moderately noisy hospital it would be between 50 and 60 dB (A), and in a noisy environment it would be between 60 and 70 dB (A).^1^

**Figure 1:**
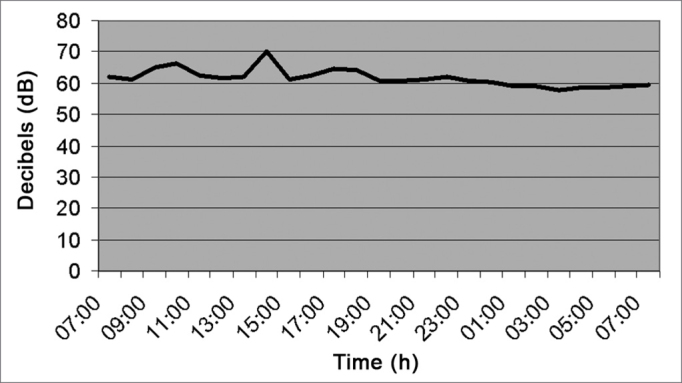
Noise intensity in the E.R. Reception desk - March/2005.

As depicted in Figure 1 (Emergency Room Reception area), the noise level was kept at a mean value of 64.2 dB (A), because it is a place with a continuous flow of people. Between 2pm and 3pm, there was a higher peak of decibels, caused by the door bell.

**Figure 2:**
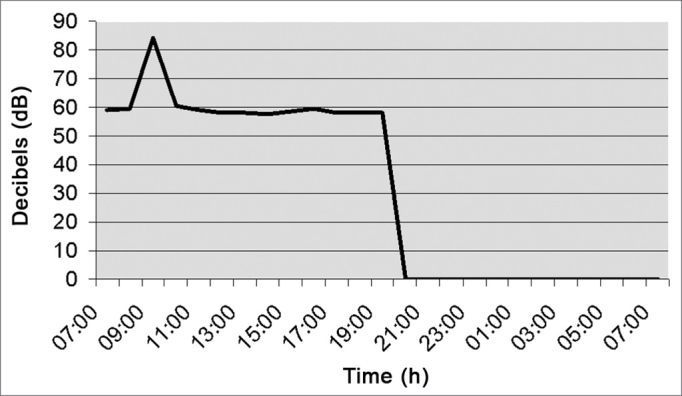
Noise intensity in the orthopedics cast room - March/2005.

Figure 2 (orthopedic cast room) depicts noise level at a mean value of 60.6 dB (A). Between 9am and 10am, there was an increase in decibel level because the cast saw was on; and from 9pm to 7am there was no work shift.

**Figure 3:**
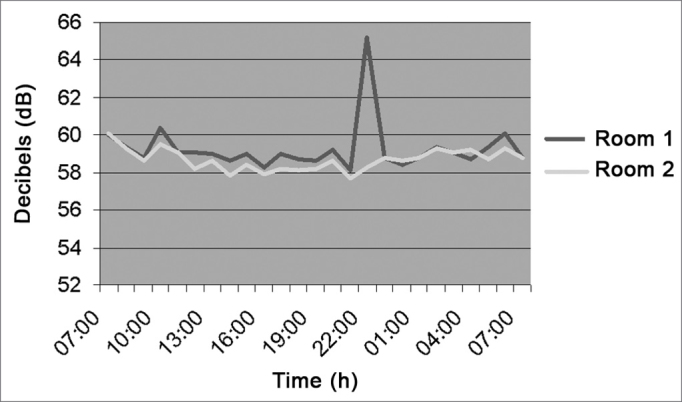
Noise intensity in the Neonatal ICU - March/2005.

Figure 3 (Neonatal ICU), shows that the noise level was of 61.4 dB(A) in average, caused by staff talk; between 10pm and 1am there was a higher decibel peak because of babies crying and monitors' alarms. Recommended levels in nurseries are of 35 to 45 dB (A), in accordance to the ABNT.

As far as nursing activities are concerned in the neonatal ICU, we observed the evolutional process of the premature newborn physiological functions. In this context we had the fragile neonates, in severe conditions in the incubator, in an environment of much mayhem and noise, such as staff conversations, alarms from devices and a high frequency of medical procedures, which are all visibly uncomfortable for the baby.^8^

Noise prevention is something that has to start before the acquisition and installation of equipment, or handling of these, since later changes may be more costly. Some noise sources, such as the unavoidable use of oxygen, suction equipment or aspirators that can not be changed; nonetheless, the alarms could be quieter, especially during the night shift.^4^

The stress produced by the hospital environment and technical procedures cause physiological changes to the newborn, such as apnea, bradicardia, reduction in oxygen partial pressure (PO2), increase in caloric demand, thus making it difficult for these neonates to gain weight; and moreover, it impairs their neurological development.^8^

**Figure 4:**
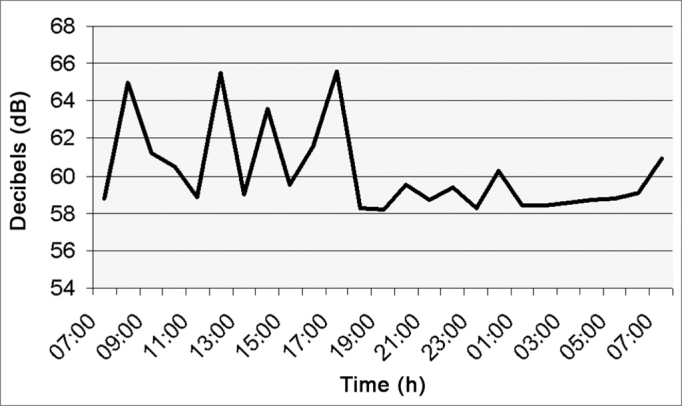
Noise intensity in the Hospital ICU - March/2005.

Figure 4 (ICU) has a noise level mean value of 62.7 dB (A), varying between 58 and 65dB (A) from 7am to 7pm because of staff conversations and equipment with sound alarms.

Numerous studies have shown that sleep deprivation is a common problem in ICU, thus impairing sleep quality, and consequently these patients have less REM sleep. In such settings, it would be desirable to significantly reduce the sound level, especially during the night.^1^

Noise control in hospitals is considered a priority; thus, we must insist in studies that show the excessive exposure to which these people are subject to, staff and patients, in the ICU, in order to prevent sound pollution, and foster staff participation in reducing noise and enhancing comfort in these areas. Simple actions, such as closing the doors, speaking softly, may dramatically reduce sound levels.^9^

**Figure 5:**
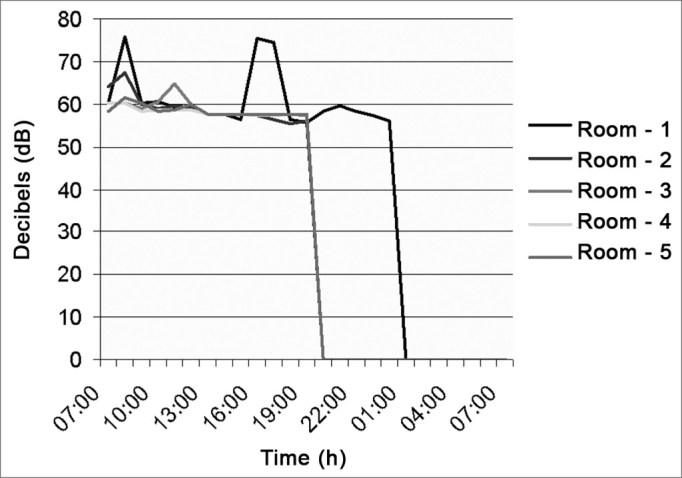
Noise intensity in the Surgery Center - March/2005.

Figure 5 (surgical center) presents five surgical rooms in which the noise level was of 59.1 dB (A) in average. Only surgical rooms 1, 2 and 3 had a higher decibels peak because they held femur fracture surgery with a team of professionals and power equipment such as power drills. From 2am to 7am the surgical center only provides care in an emergency basis.

**Figure 6:**
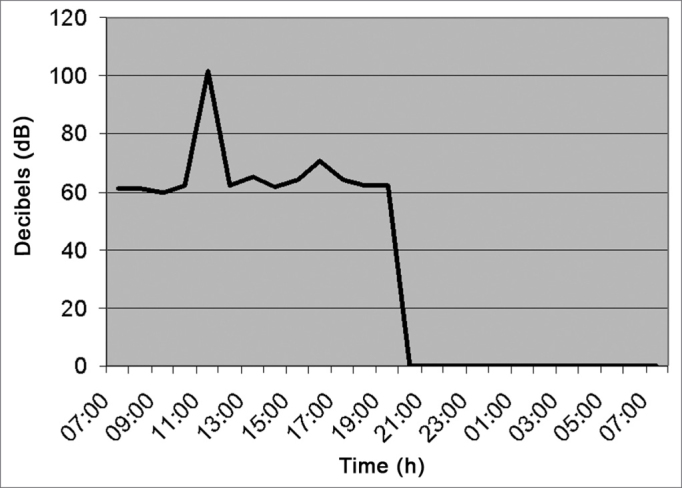
Noise intensity in the Sterilization Center - March/2005.

Figure 6 (sterilization center) shows an average of 66.0 dB(A), and between 11am and 1pm there was a 100 dB(A) relevant peak, because at this time there were equipment at work such as the autoclave and the air compressor.

**Figure 7:**
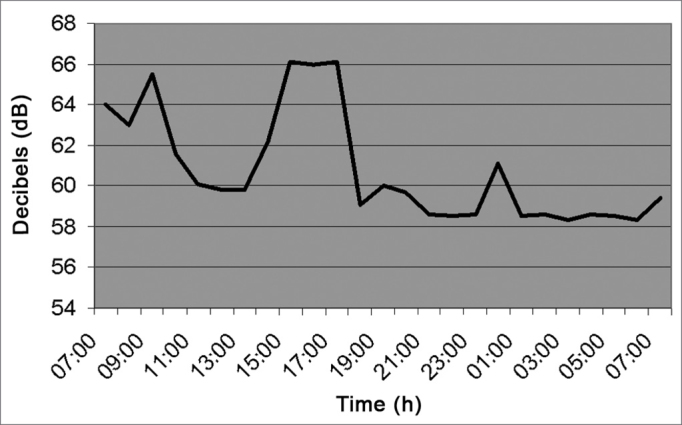
Noise intensity in the Hospital Pharmacy - March/2005.

As we can see in Figure 7 (Pharmacy), the noise level varied between 7am and 7pm, going from 58 to 66dB, with mean value of 63.3 dB(A) because the pharmacy never closes. Between 7am and 5pm, there were some decibel peaks caused by staff and the kitchen exhaust which is installed right next to the pharmacy, and when activated increases the noise level in this sector.

**Figure 8:**
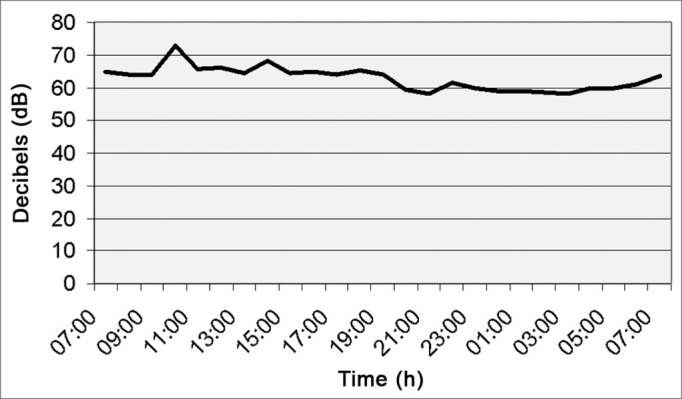
Noise intensity in the Pediatrics department (nurse station) - March/2005.

As we see in Figure 8 (pediatrics), the noise level between 7am and 7pm of the following day was kept around 6060 dB (A) because in pediatrics there is a relevant flow of children and staff. Between 10 am and 11am, there was a peak of 70dB (A) caused by conversation and children crying.

**Figure 9:**
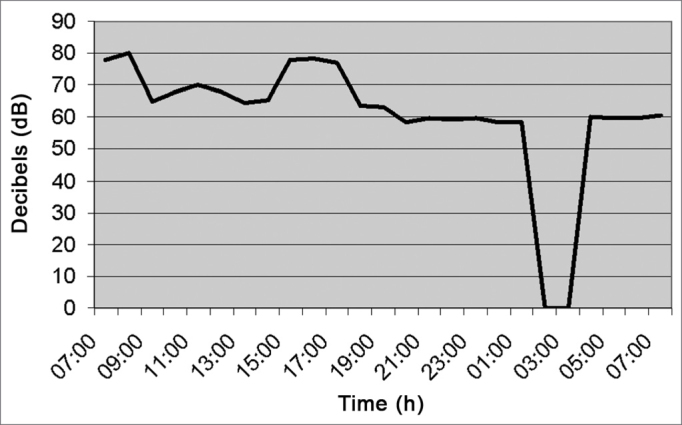
Noise intensity in the Hospital Kitchen - March/2005.

Figure 9 (kitchen) shows average noise level of 62.9 dB (A) because two exhausts were on there. From 2am to 4am it was closed, and went back to work at 5am.

**Figure 10:**
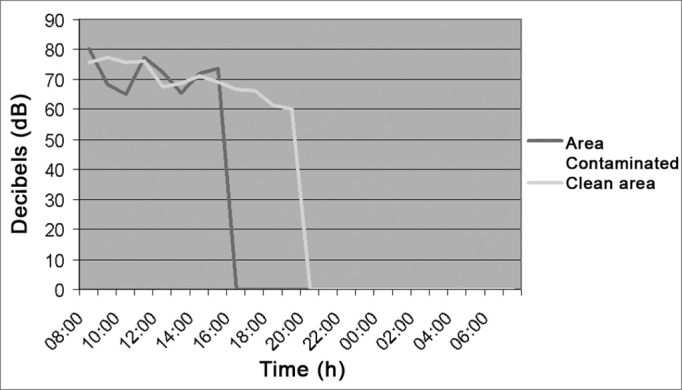
Noise intensity in the Laundry - clean area / contaminated area - March/2005.

Figure 10 shows an average of noise intensity in the laundry of 71.5 dB (A) considering both, the clean and the contaminated areas.

Mendoza-Sánchez et al., 1996, noticed noise levels between 50 and 59 dB(A) in a place considered moderately noise; except for the ICU, where noise levels went above 59 dB(A), because of different devices such as monitors, continuous infusion pumps, mechanical ventilation devices, alarms and other noise sources.^10^ These values make us consider as high the values found for this sector by this study, daily averages were of 66.30 (dB), and the equipment they list may also be associated with noise generation. Besides the equipment, the staff talking about patient care or even engaged in personal conversations may increase noise levels in this environment.

Studies carried out on the relationship between noise level, exposure duration and hearing loss tells us about the importance of sound monitoring within a program of Hearing Protection. Exposure to high sound levels may be very harmful, totally harmless or something between these two limits. The key issue here is exposure duration, which determines the impact noise exposure has on the human hearing. Noise exposure level is determined by means of sound measurements, involving sound pressure and time. Therefore, it is a parameter difference from noise levels existing in plant floors, which does not depend on exposure duration.^11^

## CONCLUSION

In all the analyzed sectors, the noise level found in this hospital is considerably above recommended values.

There was no significant difference between night and day exposure levels, despite a mild trend for less noise during the night shift.

By analyzing the data from the present study we notice the need to alert the population about the risks of noise exposure those hospitalized patients are taking, as well as those individuals who work in these hospitals, where, theoretically, there should be greater awareness.^7^

The hospital team should be more aware of noise and its effects, so that they may act more efficiently in reducing noise pollution. Thus, we believe that hospital sectors will become more silent and calm, benefiting patients and staff alike.

## References

[1] Pereira RP, Toledo RN, Amaral JLG, Guilherme A (2003). Qualificação e quantificação da exposição sonora ambiental em uma unidade de terapia intensiva geral.. Rev Bras Otorrinolaringol.

[2] Fernandes M, Morata TC (2002). Estudo dos efeitos auditivos e extra-auditivos da exposição ocupacional a ruído e vibração.. Rev Bras Otorrinolaringol.

[3] Dias A, Cordeiro R, Conente JE (2006). Gonçalves CGO.. Associação entre perda auditiva induzida pelo ruído e zumbidos. Cad de Saúde Pública.

[4] Tsiou C, Eftymiatos D, Theodossopoulou E, Notis P, Kiriakov K (1998). Noise Sources and levels in the Evgenidion Hospital intensive care unit.. Intens Care Med.

[7] Leme OLS (2001). Estudo audiométrico comparativo entre trabalhadores de área hospitalar expostos e não-expostos a ruído.. Rev Bras Otorrinolaringol.

[8] Correa LC, Zago SBAM, Posso SBM, Criollo TC (2004). Mapeamento de estudos sobre o risco potencial do ruído em neonatos internados em unidade de cuidados intensivos.. Rev Univap.

[9] Grumet GW (1988). MD. Sounding board: Pandemonium in the modern hospital.. N Engl 3 Med.

[10] Mendoza-Sánchez RS, Roque-Sánchez RH, Moncada-González B (1996). Nível de ruído en una institución hospitalaria de assistencia y docencia.. Gac Méd Méx.

[11] Nepomuceno JA, JA Nepomuceno, AA Nudelmann, EA Costa, Seligman J, RN Ibñez (1995). Perda auditiva induzida pelo ruído (PAIR): ed..

